# Multi-omics study identifies that *PICK1* deficiency causes male infertility by inhibiting vesicle trafficking in Sertoli cells

**DOI:** 10.1186/s12958-023-01163-w

**Published:** 2023-11-25

**Authors:** Jing Jin, Kaiqiang Li, Yaoqiang Du, Fang Gao, Zhen Wang, Weixing Li

**Affiliations:** 1 Laboratory Medicine Center, Zhejiang Center for Clinical Laboratories, Zhejiang Provincial People’s Hospital (Affiliated People’s Hospital), Hangzhou Medical College, Hangzhou, 310000 Zhejiang China; 2 Laboratory Medicine Center, Allergy Center, Department of Transfusion Medicine, Zhejiang Provincial People’s Hospital (Affiliated People’s Hospital), Hangzhou Medical College, Hangzhou, 310000 Zhejiang China; 3 Center for Reproductive Medicine, Department of Reproductive Endocrinology, Zhejiang Provincial People’s Hospital (Affiliated People’s Hospital), Hangzhou Medical College, Hangzhou, 310000 Zhejiang China

**Keywords:** Male infertility, *PICK1* truncating mutation, Sertoli cell, Vesicle trafficking, Biomarker

## Abstract

**Background:**

Infertility affects approximately 10–15% of reproductive-age men worldwide, and genetic causes play a role in one-third of cases. As a Bin-Amphiphysin-Rvs (BAR) domain protein, protein interacting with C-kinase 1 (PICK1) deficiency could lead to impairment of acrosome maturation. However, its effects on auxiliary germ cells such as Sertoli cells are unknown.

**Purpose:**

The present work was aimed to use multi-omics analysis to research the effects of *PICK1* deficiency on Sertoli cells and to identify effective biomarkers to distinguish fertile males from infertile males caused by *PICK1* deficiency.

**Methods:**

Whole-exome sequencing (WES) was performed on 20 infertility patients with oligozoospermia to identify pathogenic *PICK1* mutations. Multi-omics analysis of a *PICK1* knockout (KO) mouse model was utilized to identify pathogenic mechanism. Animal and cell function experiments of Sertoli cell-specific *PICK1* KO mouse were performed to verify the functional impairment of Sertoli cells.

**Results:**

Two loss-of-function deletion mutations c.358delA and c.364delA in *PICK1* resulting in transcription loss of BAR functional domain were identified in infertility patients with a specific decrease in serum inhibin B, indicating functional impairment of Sertoli cells. Multi-omics analysis of *PICK1* KO mouse illustrated that targeted genes of differentially expressed microRNAs and mRNAs are significantly enriched in the negative regulatory role in the vesicle trafficking pathway, while metabolomics analysis showed that the metabolism of amino acids, lipids, cofactors, vitamins, and endocrine factors changed. The phenotype of *PICK1* KO mouse showed a reduction in testis volume, a decreased number of mature spermatozoa and impaired secretory function of Sertoli cells. In vitro experiments confirmed that the expression of growth factors secreted by Sertoli cells in *PICK1* conditional KO mouse such as Bone morphogenetic protein 4 (BMP4) and Fibroblast growth factor 2 (FGF2) were decreased.

**Conclusions:**

Our study attributed male infertility caused by *PICK1* deficiency to impaired vesicle-related secretory function of Sertoli cells and identified a variety of significant candidate biomarkers for male infertility induced by *PICK1* deficiency.

**Supplementary Information:**

The online version contains supplementary material available at 10.1186/s12958-023-01163-w.

## Introduction

Infertility affects approximately 10–15% of reproductive men worldwide [1], and in China, more than 20 million men of childbearing age suffer from infertility. Male infertility has high phenotypic heterogeneity, from complete loss of sperm to obvious changes in sperm quality [2], among which oligoasthenospermia accounts for approximately three-quarters of sterile males [3]. The cause of infertility includes abnormalities of the immune and endocrine systems, infection, lack of trace elements, and genetic causes, which play a role in one-third of the cases [4]. Genetic testing has gradually been incorporated into the clinical guidelines and consensus on male reproduction [5]. Unfortunately, the diagnosis rate based on genomics testing is relatively low, accounting for only 10–15% of infertility patients [6]. Currently, with the rapid development of detection technology for in vitro diagnostics, multi-omics analysis integrating genetics and metabolism is of great significance to the precise diagnosis and personalized treatment of infertility, which is expected to assist in ART-related treatment decisions [7, 8]. However, the application of multi-omics research in the field of male infertility is still rare, and the integration of omics data is still relatively limited.

Protein interacting with C-kinase 1 (PICK1) is a multifunctional binding protein that is widely distributed in the nervous and reproductive systems, with abundant expression in the brain and testis [9]. It has been reported to be related to neuroendocrine processes, the secretion of insulin and the development of tumors [10, 11]. PICK1 is located in immature secretory vesicles in the cytoplasm, and the importance of PICK1 in the transport of Golgi-derived vesicles has attracted much attention recently. Its lipid binding domain Bin-Amphiphysin-Rvs (BAR) plays a key role in the secretion of growth hormone, promoting the formation of dense core vesicles [12, 13]. In the male reproductive system, PICK1 is involved in the transport of precursor particles in dense core vesicles in spermatocytes [14]. In *PICK1* knockout (KO) mouse, the preacrosomal vesicles in sperm cells are scattered in the cytoplasm, without the formation of mature acrosomes, which indicates that the acrosome formation disorder in *PICK1* KO mouse may occur in the Golgi stage [15]. Previous studies have confirmed that PICK1 is vital in the normal development of sperm [14]. However, its role in auxiliary germ cells such as Sertoli cells is still ambiguous [16].

Sertoli cells are distributed around germ cells, and their various secretory factors accelerate the differentiation and maturation of spermatogonial stem cells [17]. Sertoli cells are crucial for spermatogenesis in the seminiferous epithelium because their actin cytoskeleton supports vesicular trafficking, cell junction formation, protein anchoring, and spermiation. In patients with nonobstructive azoospermia, the secretion of inhibin B, Androgen receptor (AR), Bone morphogenetic protein 4 (BMP4), Stem cell factor (SCF) and other growth factors secreted by Sertoli cells is significantly reduced [18]. The secretions of Sertoli cells are synthesized in the Golgi apparatus. Therefore, abnormal transport of Golgi vesicles in Sertoli cells may result in stagnation of spermatogenesis, leading to disorders in the differentiation and maturation of sperm and even azoospermia [19–21]. To date, only a few molecular markers of Sertoli cells, such as inhibin B and follicle-stimulating hormone, have been used to predict the spermatogenic function of the testis [22, 23]. Therefore, an in-depth understanding of the regulation by Sertoli cells in spermatogenesis and enhancing the application of new biomarkers in the clinic are of great significance.

In this study, two rare deletion mutations in *PICK1* were identified in infertility patients by Whole-exome sequencing (WES). Coincidentally, the inhibin B levels of these patients decreased significantly, indicating that the secretory function of Sertoli cells is destroyed. Systematic multi-omics analysis of *PICK1* knockout mouse indicated that infertility caused by PICK1 deficiency could be attributed to vesicle secretion dysfunction in Sertoli cells. Furthermore, cytology and animal experiments of Sertoli cell-specific *PICK1* knockout mouse further confirmed the impaired secretory function of Sertoli cells. Benefiting from high-throughput multi-omics analysis, potential significant biomarkers of multiple categories were screened out. In general, for the first time, we attribute male infertility caused by PICK1 deficiency to impaired vesicle trafficking function of Sertoli cells to provide a key scientific basis for the precise diagnosis and treatment of male infertility.

## Materials and methods

### Patient recruitment and whole-exome sequencing

This study was approved by the Human Ethical and Scientific Review Committees of Zhejiang Provincial People’s Hospital, and all participants gave written informed consent for the use of their peripheral blood in research. Twenty infertile males with oligoasthenospermia were enrolled in this study. The inclusion criteria included couples living together for more than one year after marriage with a normal sex life and engaged in no contraceptive measures. At the same time, the total sperm count was less than 39×10^5^ or the percentage of sperm with progressive motility was less than 40. Chromosomal abnormalities causing infertility were excluded in all subjects.

Genomic DNA was extracted from the peripheral blood of the patient according to standard procedures using the QIAGEN DNeasy Blood & Tissue Kit (Qiagen, Valencia, CA, USA). Whole-exome capture using the Agilent SureSelect Human All Exon v5 Kit (Agilent Technologies, Santa Clara, CA, USA) and high-throughput sequencing by utilizing an Illumina HiSeq 4000 sequencer (Illumina, San Diego, CA, USA) were conducted.

The Trim Galore program was used to remove low-quality reads and adapters. The filtered reads (Phred-scaled quality score ≥ 30 and read length ≥ 80 bp) were aligned to the human reference genome (GRCH37/hg19) with the Burrows‒Wheeler Alignment Tool pipeline. Picard was then utilized to realign the reads from the BAM files and label the duplicated reads. All variants, including single-nucleotide variants and indels, were called according to three incorporated genome analysis toolkit tools: RealignerTargetCreator, IndelRealigner, and BaseRecalibrator.

### Construction and identification of ***PICK1*** KO mouse

Animal ethics was approved by the Animal Protection and Use Committee of Zhejiang Provincial People’s Hospital. The male mouse used in this study were all 3–6 months old, as this is the time of optimum fertility. The experimental animals consisted of two groups: *PICK1* KO (*PICK1* –/–) male mouse and wide type (WT) male C57/BL6 (*PICK1* +/+). Male *PICK1* KO mouse were obtained by copulation of *PICK1* heterozygous genotype mouse. Sanger sequencing was used to verify that *PICK1* was knocked out. The amplification template was extracted from the toes of mouse. The primer sequences were as follows: *PICK1*-1, 5′-TCACTTGCCAGAGGAGAAAACTG-3′; *PICK1*-2, 5′-AAAAATAGGCGTATCACGAGGC-3′; *PICK1*-3, 5′-CACTCGCAG CT TGTTCTGATCTG-3′.

### cDNA library preparation and metabolomics sequencing

RNA was extracted from testicular tissue in male *PICK1* KO and WT C57/BL6 mouse, and an Agilent 2100 bioanalyzer was used to detect the content and integrity of RNA. The NEBNext® Ultra™ RNA Library Prep Kit for Illumina® was used for RNA library construction. The first strand of cDNA was synthesized in the M-MuLV reverse transcriptase system using fragmented mRNA as a template and random oligonucleotides as primers. After terminal repair, an A-tail was added to the purified double-stranded cDNA, and sequencing joints were connected to screen out approximately 250–300 bp cDNA. PCR amplification was carried out, and PCR products were purified again using AMPure XP beads to obtain the library. After qualified library inspection, Illumina sequencing was performed based on sequencing by synthesis after pooling different libraries according to the requirements of effective concentration and target on-machine data volume.

Flow-injection analysis with tandem mass spectrometry was used for the analysis of carnitine, acylcarnitines, lipids, and hexoses. We used liquid chromatography to separate amino acids and biogenic amines.

### Identification of differentially expressed microRNAs, mRNAs and metabolites

We used the DEseq2 package to normalize the RNA-seq data and performed a two-dimensional principal component analysis (PCA) and hierarchical clustering to visualize the similarities and differences between the testis tissues of *PICK1* KO infertility mouse and WT mouse. Subsequently, differential gene expression analysis was performed using DEseq2. A gene was considered a differentially expressed gene (DEG) if it met the following criteria: a false discovery rate < 0.05 and a |log2 fold change | > 0.5 in DEseq2. A volcano plot visualizing all DEGs between different subjects was constructed in R with the ‘ggplot2’ package, and a heatmap for the DEGs was drawn using the R software package ‘pheatmap’.

MetaboAnalysis4.0 was used to analyse the metabolomics data of testicular tissue in mouse, and the low-quality metabolic species that could not be detected in half of the samples were filtered. The normalization by Sum standardization method was used to obtain normally distributed data. The Variable influence on projection (VIP) values of differential metabolites at each species level were calculated to evaluate their importance in the difference between the experimental and control groups. Those with VIP > 1 and *P* value < 0.05 were selected as potential differential metabolites.

### qRT-PCR analysis of differentially expressed microRNAs

miRNA quantifcation: Bulge-looprM miRNA qRT-PCR PrimerSets (one RT primer and a pair of qPCR primers for each setspecific for miR-300-3p, miR-182-5p, miR-337-5p, let-7a-1-3p, miR-470-5p and miR-18a-5p are designed by RiboBio (Guangzhou, China). The total RNA was extracted with TRIzol Reagent. Then, miRNA bulge-loop was reverse transcribed with the First-Strand cDNA Synthesis Kit from RIBBIO (Guangzhou, China) and quantified by qPCR using SYBR Green Real-Time PCRMaster Mix Kit from Vazyme (Nanjing, China). U6 was utilized as an intemal control for miRNAs.

### Enrichment analysis by gene ontology, kyoto encyclopedia of genes and genomes and construction of a physical interaction network

The targeting relationship between different miRNAs and genes was predicted by the biological prediction websites miRanda and RNAhybrid [28, 29]. To further research the function of the DEGs and target genes of differentially expressed miRNAs, biological processes (BP) analysis of gene functional analysis was conducted on the David website. Kyoto Encyclopedia of Genes and Genomes (KEGG) analysis of DEGs was performed by the ClusterProfiler R package. The Bonferroni step-down method was used for multiple corrections with a threshold of 0.05 for corrected *P* values.

The direct protein–protein interaction (PPI) data set used in this study was downloaded from the STRING database. We extracted the interaction data of the 150 top DEGs in the STRING database to construct an internal interaction network. To evaluate the significance of the observed network, we performed random simulations of 100,000 iterations for genes and their connections selected from the STRING database.

### Correlation analysis of biomarkers identified by multi-omics analysis

Construction and topology analysis of the competing endogenous RNAs (ceRNA) regulatory network: TargetScan was used to predict the candidate miRNAs corresponding to hub mRNAs, and LncBase was used to predict hub lncRNAs according to hub miRNAs. The lncRNA‒miRNA-mRNA network was constructed using the interaction using Cytoscape software.

Correlation study of transcriptome and metabolome: Pearson coefficient between differential genes and differential metabolites was calculated, and the heatmap of correlation between them was constructed, indicating that differential genes play a direct or indirect regulatory role in the key metabolism of the occurrence and development of diseases.

### Construction of a Sertoli cell-specific ***PICK1*** conditional KO mouse model

*PICK1*^flox/flox^ conditional KO mouse were designed to hybridize with AMH-Cre mouse to obtain Sertoli cell-specific *PICK1* KO (CKO) mouse to verify the role of PICK1 in Sertoli cell regulation of sperm differentiation and maturation. *PICK1*^flox/flox^ mouse model was provided by Nanjing Institute of Biomedicine. Then, the AMH-Cre: *PICK1*^loxp+/−^ mouse were bred with each other to generate the final CKO mouse (AMH-Cre: *PICK1*^loxp/loxp^). The specific process of construction can be found in Fig. S1.

### Isolation and culture of Sertoli cells

Sertoli cells were isolated from the testes of adult mouse. The testicular tissue was cut into 1 mm^3^ pieces and centrifuged with Hank’s equilibrium salt solution to remove blood cells. First, digestion was carried out by shaking with 0.25% trypsin and 20 µg/mL DNAse in a water bath at 37 ℃ for 30 min. The digestion was terminated by Hank’s solution. After centrifugation at 1 000 r/min, the precipitated tissue was taken. The precipitated tissue was then oscillated and digested with 0.2% collagenase, 15 µg/mL DNAse and 0.15% hyaluronidase in a water bath at 37 ℃ for 45 min, filtered with a 200-mesh filter, and centrifuged at 1 000 r/min for 3 min, and the supernatant was removed. The precipitated cells were added to DMEM/F12 culture medium and stored in a 37 °C, 5% CO2 and saturated humidity incubator. The number of Sertoli cells was counted, and the cell concentration was adjusted to 1 × 10^6^ cells/ml. Its purity was verified by immunofluorescence staining with the Sertoli cell-specific antibody GATA.

## Results

### ***PICK1*** deletion mutations cause dysfunctional secretion by Sertoli cells

**Fig. 1:**
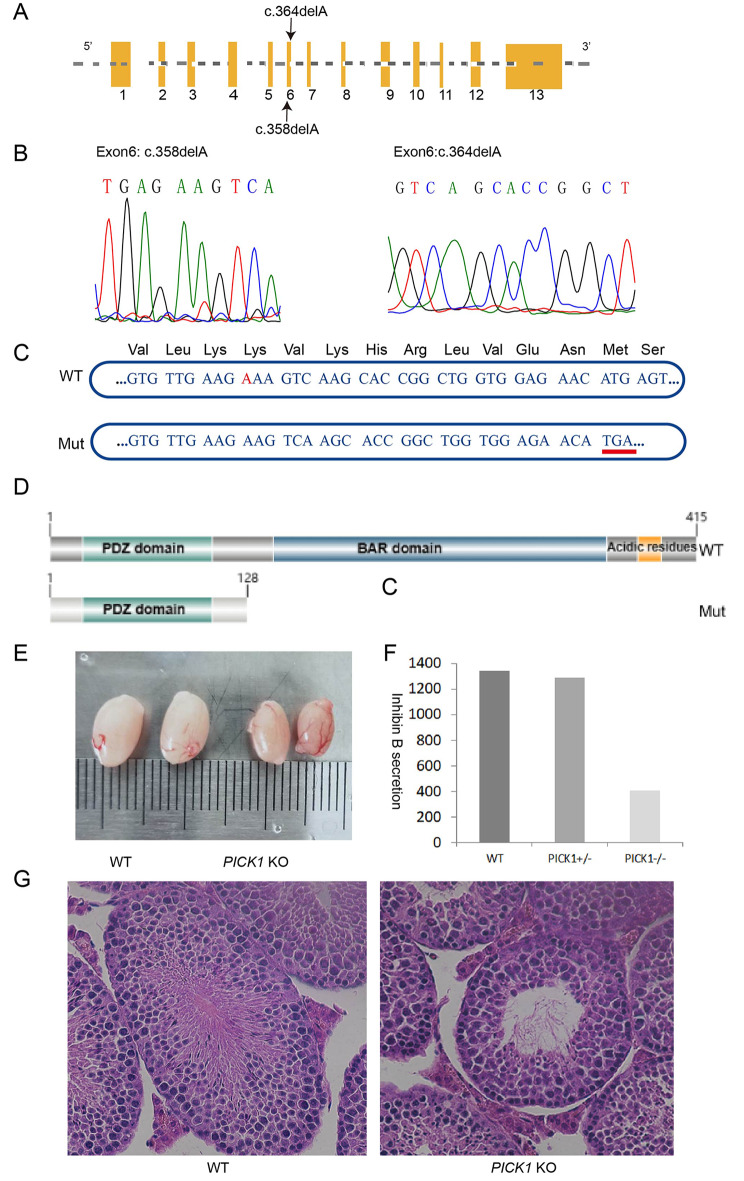
*PICK1* loss-of-function deletion mutations in male infertility patients with oligoasthenospermia. **(A)** Schematic of *PICK1*. Yellow boxes represent the coding exons; black arrow indicates the mutation identified in this study. **(B)** Verification of c.358delA and c.364delA mutations by Sanger sequencing. **(C)** The c.358delA mutation caused the stop codon. **(D)** The truncation of the PICK1 protein caused by c.358delA mutation. **(E)** The testicles of *PICK1* KO mouse were significantly smaller than those of wild-type mouse. **(F)** The level of inhibin B secreted by Sertoli cells was the lowest in *PICK1* KO mouse and was lower than that in heterozygous mouse and wild-type mouse. **(G)** HE staining at 20X magnification showed that the lumen of seminiferous tubules was enlarged and germ cells were reduced

PICK1 has been reported to be localized to Golgi-derived proacrosomal granules. We applied whole-exome second-generation sequencing to analyse mutations in the coding regions of 20 patients with oligozoospermia. After filtering out mutations with allele frequencies greater than 0.1% through public databases and 14 common methods and software such as PolyPhen2, SIFT, LRT, CADD to predict conservativeness and deleteriousness of mutant loci, two deletion mutations were finally identified in the *PICK1* gene (Fig. 1A). The two mutations detected in oligozoospermia patients in this study cannot be find in the recognized exon database such as 1000 Genomes, ESP6500 and ExAC, indicating that the two mutations are extremely rare. The two deletion mutations c.358delA and c.364delA in coding sequences were confirmed by Sanger sequencing (Fig. 1B). Nucleotide sequence diagram showed that c.358delA deletion mutations led to a premature stop codon (Fig. 1C). According to the protein schematic of PICK1, the premature termination codon resulted in a mutant protein length of only 128 amino acids, 233 amino acids less than the normal PICK1 protein (Fig. 1D). It is obvious that the BAR functional domain, which is closely related to vesicle secretion, cannot be transcribed.

Clinical tests have shown that infertile men carrying *PICK1* deletion mutations have a specific decrease in serum inhibin B levels secreted by Sertoli cells, which is synthesized in the Golgi apparatus. From this specific clinical picture, we hypothesized that *PICK1* deficiency may cause a defect in the secretory function of Sertoli cells. To investigate the pathological mechanisms in depth, we constructed KO mouse to carry out biological function experiments. Initially, the testes of the KO mouse were found to be significantly smaller than those of the wild-type mouse (Fig. 1E). The total sperm counts of *PICK1* KO mouse [mean=(9.533 ± 0.29)×10^5^] were significantly decreased compared to WT mouse [mean=(24.13 ± 3.13)×10^5^ ] (Fig.S2A). Breeding experiments showed that the reproductive ability of the KO mice was negatively affected (Fig.S2B). In addition, deformities were observed in the sperm heads of the KO mice under electron microscopy (Fig.S2C). Compared to WT and *PICK1* heterozygous genotype mouse, serum inhibin B levels in *PICK1* KO mouse were significantly reduced (Fig. 1F). Immunofluorescence staining showed that seminiferous tubules contained fewer sperm, thinner epidermis, and empty cavities (Fig. 1G). Animal experiments have preliminarily demonstrated that *PICK1* deficiency can lead to impaired secretory function of auxiliary germ cells and an abnormal reproductive system.

### Targeted genes of differentially expressed microRNAs are significantly enriched in the vesicular trafficking functional pathways

Due to the complexity of disease etiology, multi-omics approaches have been applied to a wide range of biological problems based on data integration across omics layers and network modelling. To investigate the pathophysiological mechanisms of impaired reproductive function in *PICK1* KO mouse, we performed multi-omics high-throughput sequencing of testicular tissue and performed integrated analysis.

**Fig. 2:**
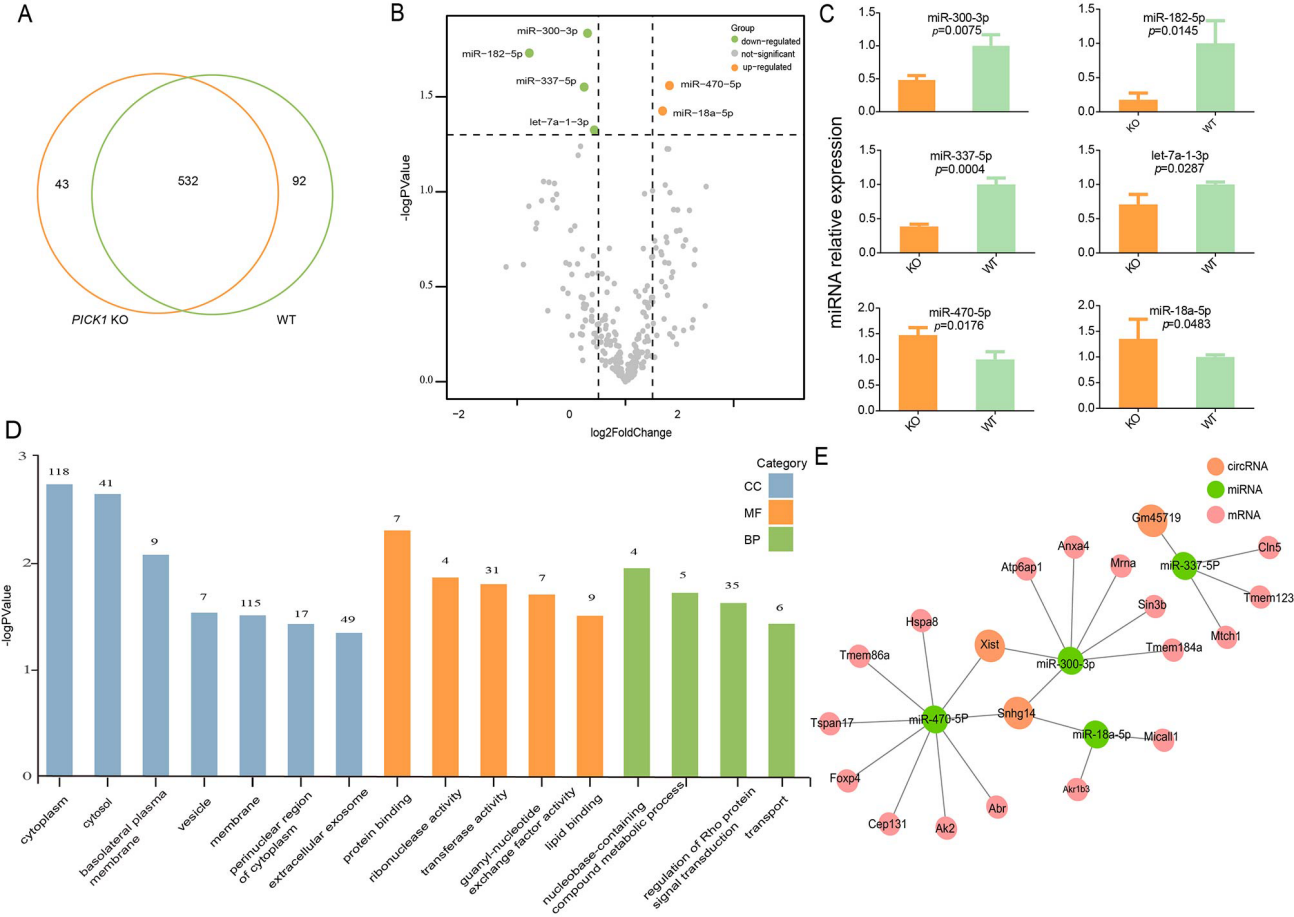
Comprehensive transcriptome analysis of microRNA and gene ontology enrichment analysis of targeted genes. **(A)** Venn diagram of microRNAs detected in PICK KO mouse and WT mouse. **(B)** Volcano plot highlighting differentially expressed microRNAs in oligoasthenospermia patients. The orange dots illustrate the 3 upregulated DEGs, while the green dots illustrate the 3 downregulated DEGs (adjusted P < 0.005 and log2 FC > 0.5). **(C)** Comparisons of the expression of the 6 microRNAs in the experimental and control groups by qPCR. **(D)** Gene ontology enrichment analysis of target genes confirmed multiple significant terms associated with vesicle trafficking. **(E)** The regulatory network of differentially expressed circRNAs, microRNAs and genes according to the transcriptome sequencing data

In the last two decades, the roles of miRNAs in development and disease have made miRNAs attractive tools and targets for novel therapeutic approaches. The number of shared annotated miRNAs between the *PICK1* KO group and the WT group was 532, with 43 miRNAs specifically identified in the KO group and 92 in the WT group (Fig. 2A). We finally identified 6 miRNAs with statistically significant differences (P ≤ 0.05, |log2FC|≥0.5) between the two groups, with miR-300-3p, miR-182-5p, miR-337-5p and let-7a-1-3p decreased in expression, while miR-470-5p and miR-18a-5p increased in expression (Fig. 2B). Subsequently, we compared the expression of these six miRNAs in the experimental and control groups by utilizing qPCR, further confirming the results based on sequencing data (Fig. 2C). Among the six differentially expressed miRNAs, the majority have been reported to be related to male reproductive function.

MiRNA mainly inhibits the expression of target genes by binding to target mRNA, promoting its degradation or hindering its translation. It is worth mentioning that the target genes of miR-182-5p, such as PRKACB, RGS17, BNC2, and SNX30, have regulatory roles in reproductive system signaling pathways. To further understand the biological function of target gene set of the six differentially expressed miRNAs, we performed an enrichment analysis of BP. The enrichment results showed multiple significant terms associated with vesicle trafficking, including ‘membrane’, ‘extracellular exosome’ and ‘transport’ (Fig. 2D). A total of 115 genes were components of ‘membrane’, and 49 targeting genes were enriched in the ‘extracellular exosome’ pathway, with a significant adjusted *P* value < 0.05. Based on the predicted target genes of miRNAs from the TargetScan database, we constructed a network of differentially expressed circRNAs, miRNAs and mRNAs (Fig. 2E).

### Differentially expressed genes play a negative regulatory role in vesicle trafficking

**Fig. 3:**
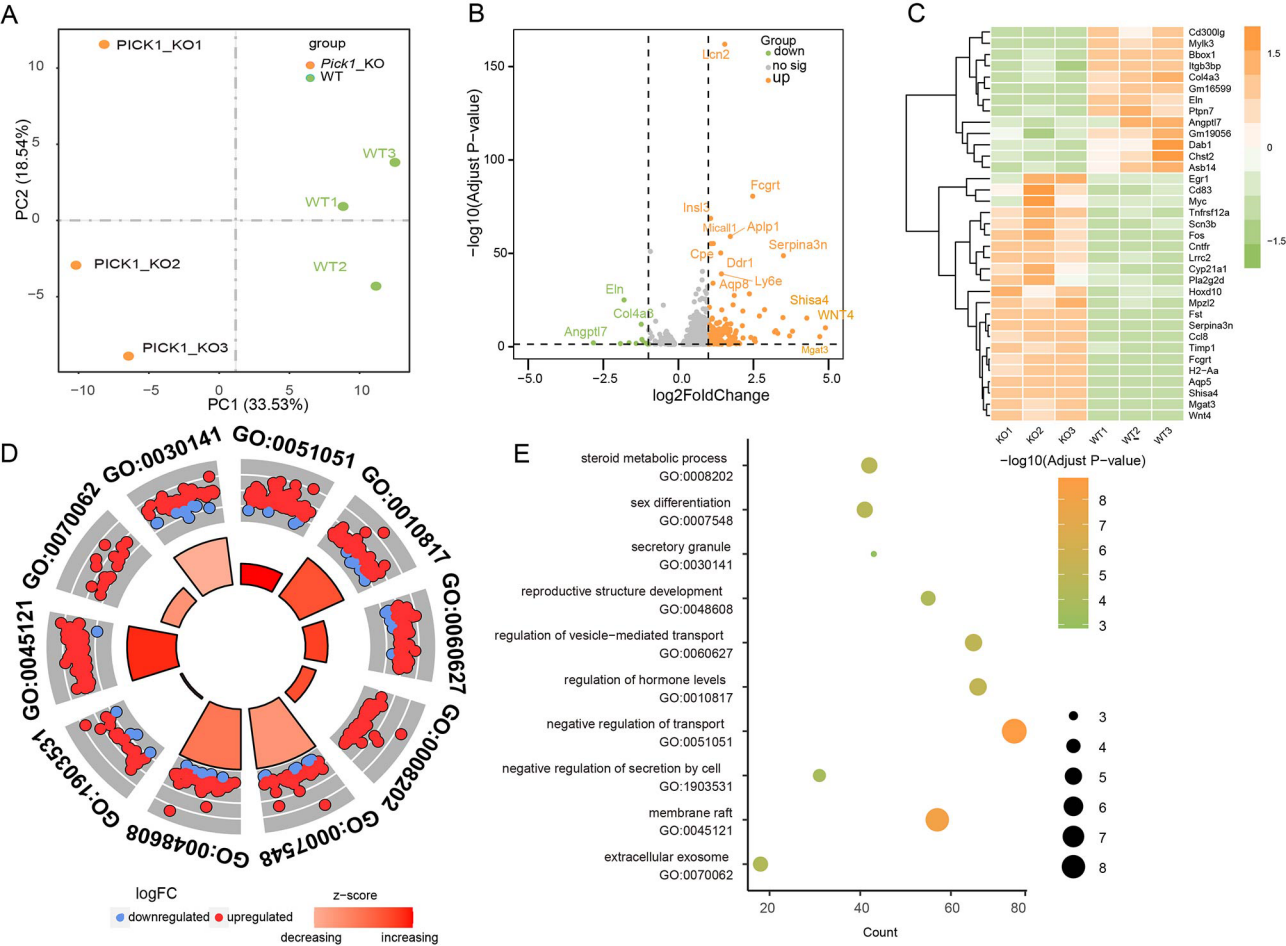
Comprehensive transcriptome analysis of mRNA and enrichment analysis of biological function. **(A)** PCA illustrating the individual differences in the RNA-seq expression profiles between the *PICK1* KO group and the control group. **(B)** Volcano plot highlighting differentially expressed mRNAs in oligoasthenospermia patients. The orange dots illustrate the upregulated DEGs, while the green dots illustrate the downregulated DEGs (adjusted P < 0.005 and log2 FCs > 0.5). **(C)** Heatmap of the top 25 DEGs. ‘Orange’ indicates high relative expression, and ‘green’ indicates low relative expression. **(D)** The top 10 most statistically significant pathways of enrichment analysis of differentially expressed genes. **(E)** The bubble diagram shows that more than half of these pathways are associated with vesicle trafficking

Quality control analysis of the raw data of the *PICK1* KO group (Fig. S3A-3D) and WT group (Fig. S3E-3H) containing the percent of bases, error rate, classification of raw reads and percent of genome regions showed that the data were qualified. Principal component analysis based on the number of transcript abundance counts revealed that the KO group and WT group could be significantly distinguished. Samples within the same group showed remarkable aggregation, indicating that the consistency within the group was excellent (Fig. 3A). The Venn diagram showed that 14,747 genes were expressed in both the KO group and WT group (Fig. S4A), and Pearson correlation analysis showed intergroup differences and intragroup consistency (Fig. S4B). The quantitative results of gene expression showed homogeneity of gene expression abundance (Fig. S4C, D).

A total of 189 DEGs (|log2(fold change) |≥0.5, Padj ≤ 0.05) were identified by the DESeq2 package after multiple hypothesis testing corrections, among which 176 were upregulated and 13 were downregulated (Fig. 3B). More than 50 DEGs are involved in the regulation of vesicle trafficking, consisting of Apoe, Alpn, Rab3b and so on. We selected the top 35 DEGs for clustering analysis to explore their expression patterns in each sample. It is intuitive that the expression of these genes is distinct between the two groups (Fig. 3C). The enrichment analysis of differentially expressed genes found that the most significant pathways were related to the development and regulation of the reproductive system, and the Z score showed that these pathways were basically upregulated (Fig. 3D). More than half of these pathways are associated with vesicle trafficking, including ‘negative regulation of transport’, ‘regulation of vesicle − mediated transport’, ‘secretory granule’, ‘negative regulation of secretion by cell’, ‘membrane raft’ and ‘extracellular exosome’ (Fig. 3E). The results fully indicated that the reproductive functions of the KO mouse, such as sex hormone metabolism and sex differentiation, were negatively affected, which was most likely due to the inhibition of vesicle trafficking of Sertoli cells and the reduction in secretory particles.

### Construction of the PPI network shows strong interaction of differential genes in reproductive system development

**Fig. 4:**
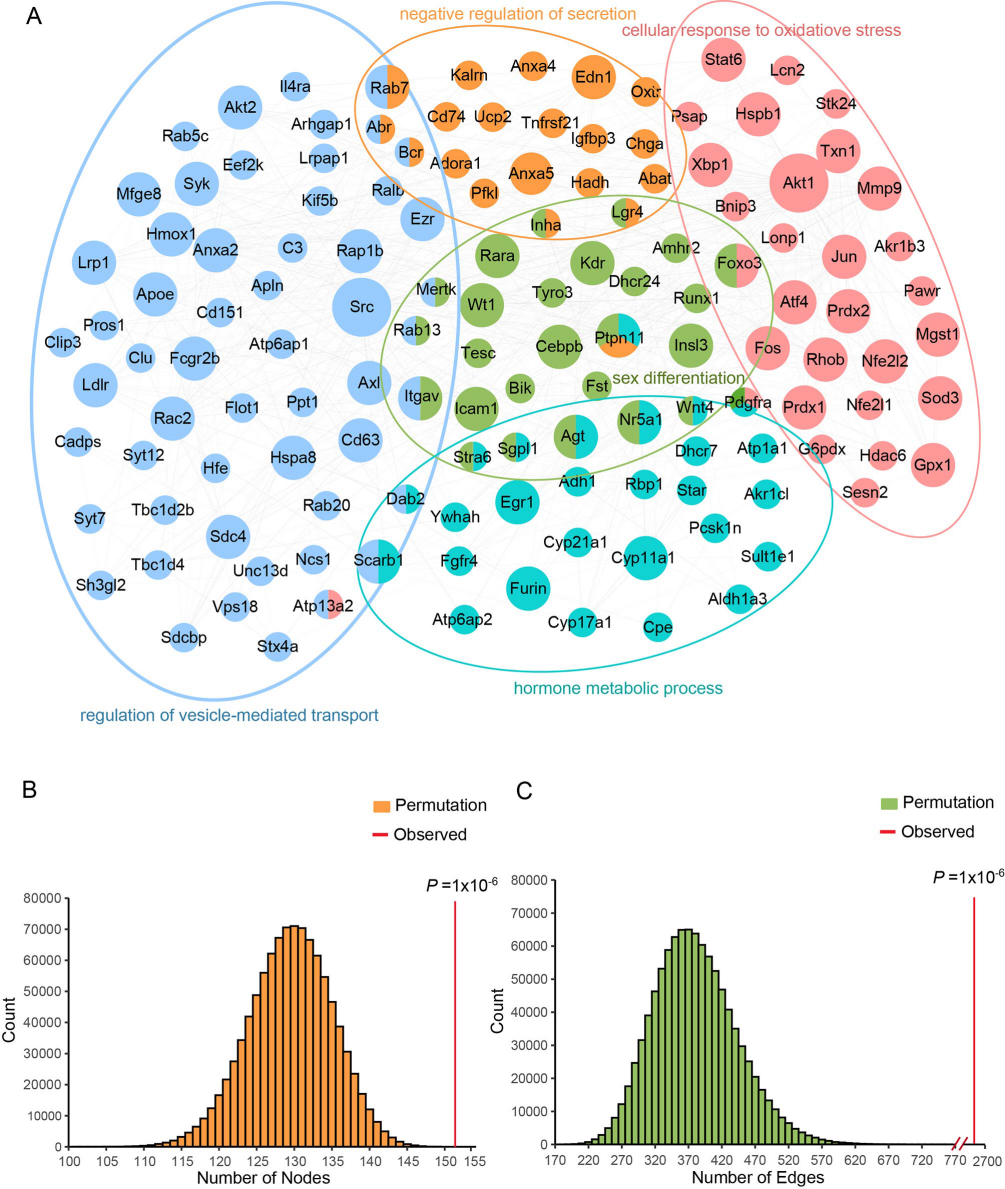
Construction of the PPI network of differentially expressed genes in reproductive system development. **(A)** PPI network of the DEGs enriched in negative regulation of secretion, cellular response to oxidative stress and regulation of vesicle trafficking. The nodes represent genes. The size of nodes indicates the number of connections. The edges denote the interactions between two genes, and the width of an edge denotes the score of a genetic interaction. **(B-C)** A permutation test of the network genes and connections was performed with 100,000 iterations

To further explore whether the differentially expressed genes were functionally associated with each other, we extracted the interaction data of the top 155 DEGs in the STRING database to construct an interconnected PPI network to evaluate their connectivity, and then Cluego was utilized to visualize the network. Consistent with the previous results, the top DEGs were clustered into 5 large categories according to their biological functions. In addition to BPs related to vesicle transport and secretion mentioned in the previous results section, or BPs directly related to reproductive system development like hormone metabolic and sex differentiation, ‘cellular response to oxidative stress’ was a newfound pathway in functional enrichment analysis (Fig. 4A). Oxidative stress has been reported as a common pathology seen in approximately half of all infertile men through damage to the sperm membrane and alteration of sperm DNA. The PPI network showed an excess number of interacting proteins among those encoded by the 155 genes (*P* = 1 × 10^− 6^) and an excess of pairwise connections (*P* = 1 × 10^− 6^) compared with 100,000 randomly simulated datasets (Fig. 4B, C). In this network, *PTPN11* is a core gene, which played multiple roles among three biological pathways. Based on the degree of genetic pathogenicity, male infertility genes were classified into three categories: ‘definitive’, ‘strong’ and ‘moderate’ [24]. To explore the relationship between *PICK1* and explicit male infertility genes, we constructed a *PICK1*-centered PPI interaction network (Fig. S5). The results showed that *PICK1* interacted with 25 definitive infertility genes and 15 strong infertility genes.

### Integrative transcriptomic and metabolomic reveals changes in vitamin, steroid hormone, and glutamine metabolism pathways

**Fig. 5:**
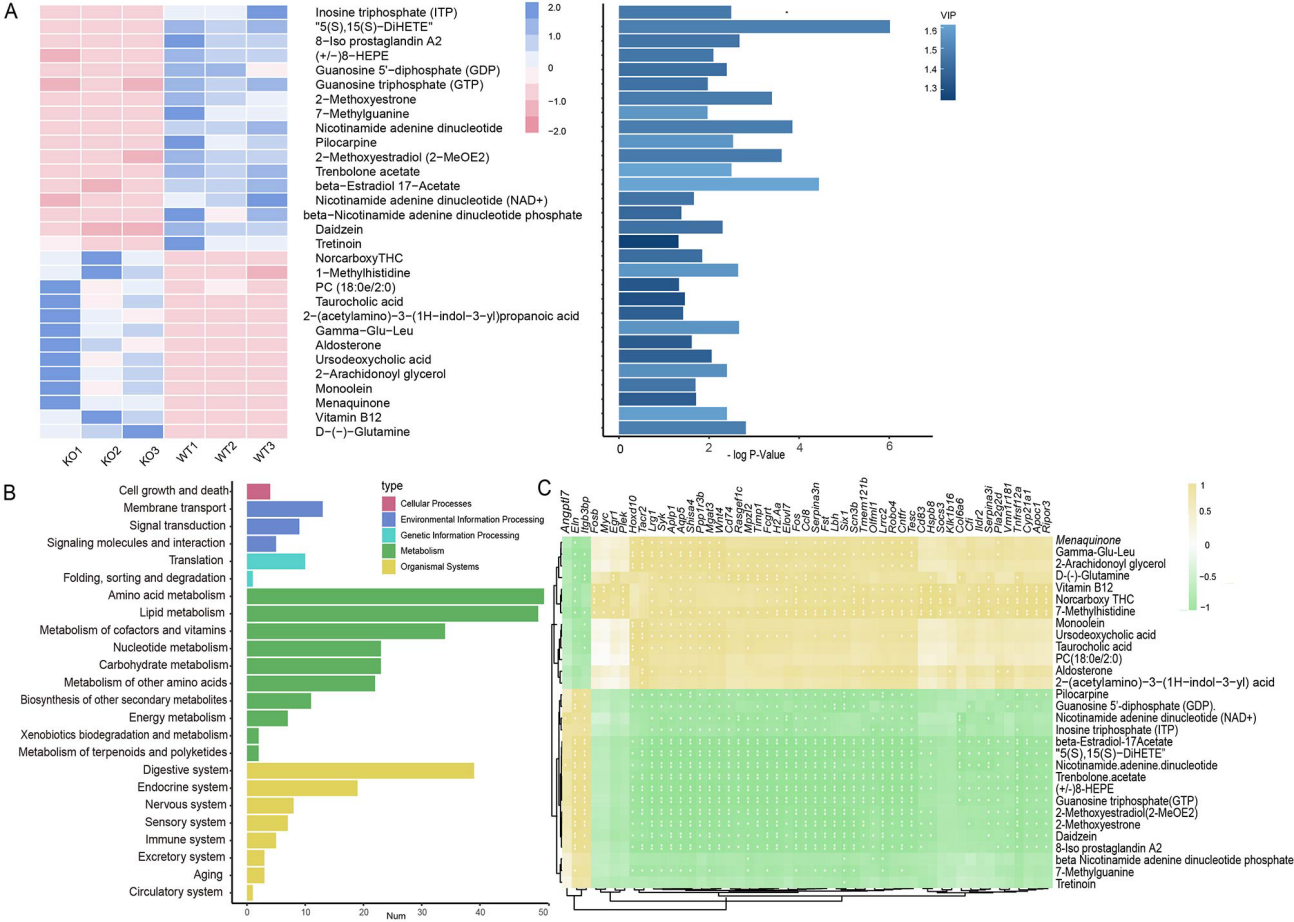
Integrative transcriptomic and metabolomic analysis. **(A)** Heatmap of the top 50 differentially expressed metabolites. **(B)** A correlation analysis heatmap of the connection between differential metabolites and DEGs was established. **(C)** The KEGG functional enrichment results showed that those differentially presented metabolites were classified as amino acid metabolism, lipid metabolism, metabolism of cofactors vitamins, endocrine system and so on

To further investigate the metabolic distinction between WT and *PICK*-KO mouse, we performed PLS-DA analyses. Multigroup variance analysis and the Kruskal‒Wallis H test was performed to identify differential metabolites. Notably, compared with the control group, D-(-)-glutamine, vitamin B12, and meaquinone (vitamin K2) were enriched in the KO group, while ITP, 5(S), 15(S)-DiHETE, and 8-isoprotaglandin A2 were significantly decreased (Fig. 5A). According to the VIP calculated in the PLS-DA model, vitamin B12 and beta-estradiol 17-acetate were the key components with significant differences (Fig. 5A). Moreover, KEGG functional annotation analysis was applied to search for the metabolic pathways. The results showed that those differentially presented metabolites were classified as amino acid metabolism, lipid metabolism, metabolism of cofactors vitamins, endocrine system and so on (Fig. 5B).

To understand the metabolic response of the testicles of *PICK1* KO sterile mouse at the transcriptome level, a correlation analysis heatmap of the connection between differential metabolites and DEGs was established (Fig. 5C). Significant correlations were found between the vast majority of differentially expressed genes and differentially expressed metabolites, indicating that differential genes play a direct or indirect regulatory role in the key metabolism of the occurrence and development of diseases.

Based on integrated multi-omics analysis, we identified a variety of significantly differentially expressed molecules that could be candidate biomarkers for male infertility induced by *PICK1* deletion (Table 1).

**Table 1:** The summary of multi-omics biomarkers identified in this study

Categories	Potential biomarkers	Log (2) Foldchange	Major biological functions have been reported
microRNA	miR-470-5p	up-regulation	inhibited mice depressive behavior through promoted hippocampal neurons growth
	miR-18a-5p	up-regulation	Modulates epithelial-mesenchymal transition in breast cancer; boost JAK2/STAT3 signaling activity to enhanced proliferation and migration of tumor
	miR-300-3p	down-regulation	potential markers for transient ischemic attack in rats
	miR-182-5p	down-regulation	possible biomarker for teratozoospermia patients in seminal plasma fluid
	miR-337-5p	down-regulation	promotes the development of cardiac hypertrophy^67^; promote lymph node metastasis of human gastric cancer
	let-7a-1-3p	down-regulation	the most abundant miRNAs family in testis; regulate the cell cycle and proliferation; associated with estrogen and androgen signaling
mRNA	C3/Rab3b/Apln	up-regulation	key regulators of membrane trafficking, regulates vesicle-mediated transport
	WNT4/Cyp21a1	up-regulation	affects the concentration of testosterone in the blood
	Atp1a1	up-regulation	regulating sertoli tight junctions and gap junction
	Ccdc39/ Cep131/ Nme5	down-regulation	required for assembly of inner dynein arms and the dynein regulatory complex and for normal ciliary motility
small Metabolite	Nicotinamide adenine dinucleotide/Menaquinone/Folic acid	up-regulation	Vitamin digestion and absorption
	Aldosterone/ 2-Methoxyestrone	up-regulation	Steroid hormone biosynthesi
	Nicotinamide adenine dinucleotide	up-regulation	regulation of AMPK signal pathway which plays vital role in sperm motility, maintenance of the integrity of sperm membranes, and the mitochondrial membrane potential
	Histamine	down-regulation	vesicle cycle in female reproduction system

### The specific cytokines secreted by Sertoli cells in ***PICK1*** conditional KO mouse were reduced

**Fig. 6:**
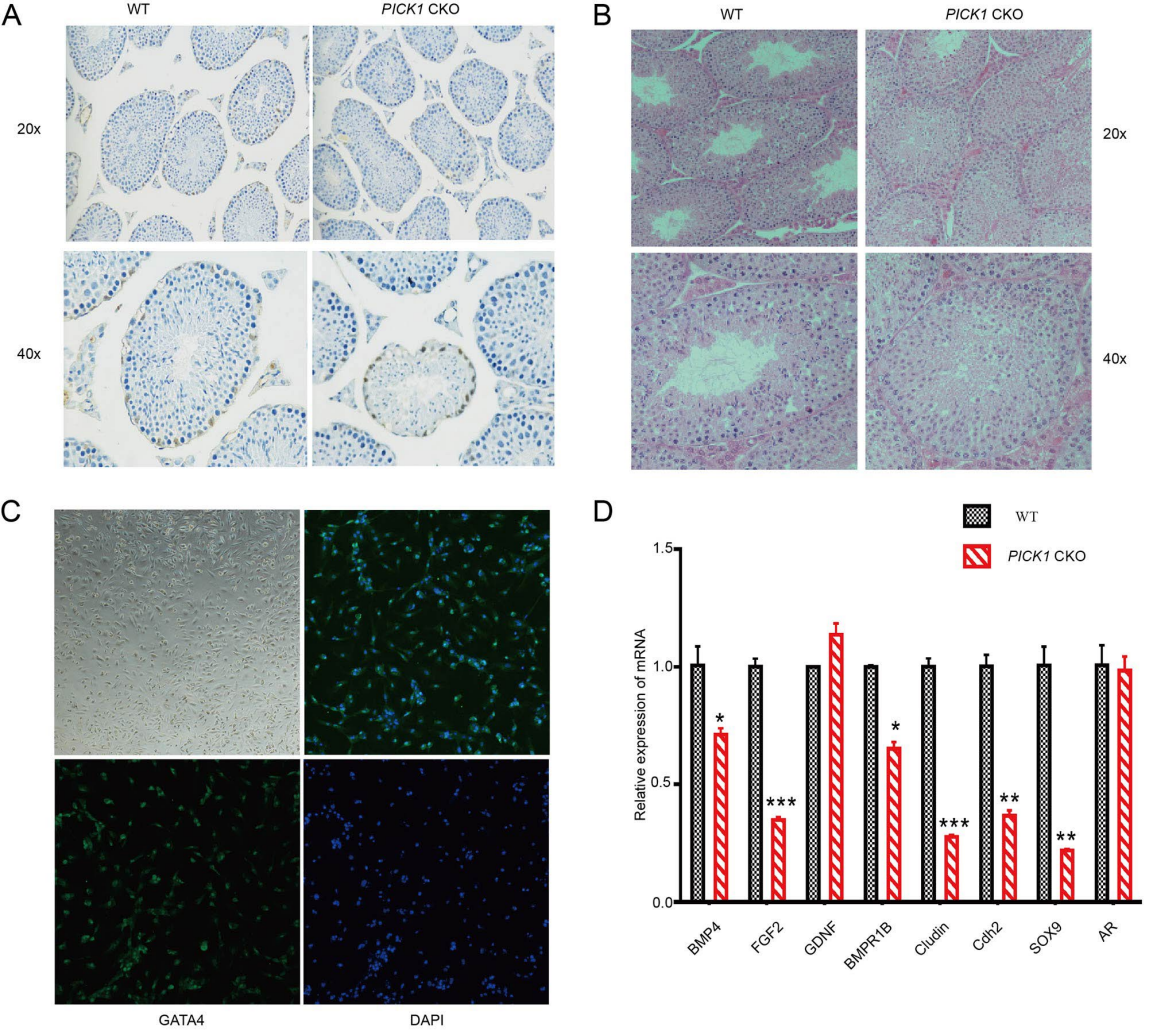
Detection of cytokines secreted by Sertoli cells in *PICK1* CKO mouse. **(A)** Immunohistochemical staining characteristics of Sertoli cells using GATA4 in *PICK1* CKO mouse and their WT littermates. **(B)** Hemate in eosin staining of testicular tissue in *PICK1* CKO mouse and their WT littermates. **(C)** Sertoli cells extracted from *PICK1* CKO mouse testis verified by the specific fluorescent antibody GATA4. **(D)** qPCR validation of cytokines specifically secreted by Sertoli cells

To further verify that secretory dysfunction of Sertoli cells caused by *PICK1* deletion plays a role in male infertility, Sertoli cell *PICK1* conditional KO mouse model was constructed and validated. GATA4, a specific marker protein of Sertoli cells, was applied in immunohistochemical staining of testes from 18-week-old *PICK1* CKO mouse and their WT littermates. We observed a significant reduction in Sertoli cells surrounding the seminiferous tubules and disorder of cell arrangement (Fig. 6A). HE staining of mouse testicular tissue showed that the number of mature sperm in the seminiferous tubule of the CKO group was significantly smaller than that of the WT group (Fig. 6B). To detect whether the secretory function of Sertoli cells is affected in *PICK1* CKO mouse, we extracted Sertoli cells from mouse testes and cultured them in vitro, which was verified by the specific fluorescent antibody GATA4 (Fig. 6C). Six of the eight cytokines specifically secreted by Sertoli cells were secreted in reduced quantities by qPCR validation, fully indicating that the secretory function of supporting cells was impaired (Fig. 6D).

## Discussion

Male infertility is a complex disease of multiple etiologies with a high degree of phenotypic heterogeneity, and genetic factors account for approximately 15% of clinical cases [5]. The pathogenic genes of male infertility fall into four categories: (1) homozygous mutations in genes that can cause cystic fibrosis and the absence of bilateral fallopian tubes [25, 26]; (2) genes associated with hypogonadism, such as NR5A1 or genes encoding steroidogenic factor [27, 28]; (3) genes encoding motor proteins, such as mutations in NAI1, DNAH5 and DNAH11 leading to dysfunction of sperm forward motor [29–31]; and (4) genes that regulate the endocrine function of auxiliary germ cells.

In this study, two deletion mutations in *PICK1* were confirmed based on WES in 20 oligasthenosperm patients, and both mutations resulted in premature protein termination truncation. PICK1 has been confirmed to be crucial for vesicle trafficking. PICK1 deficiency in sperm cells has been reported to lead to abnormal vesicle trafficking from the Golgi to the acrosome [14, 15]. The *PICK1* KO mouse model established in this study showed markedly reduced testicular volume and significantly decreased germ cells in seminiferous tubules, suggesting that *PICK1* deficiency resulting in abnormal reproductive system development is most likely the pathogenesis of oligasthenospermia. In the field of male reproduction, previous studies have focused on the role of PICK1 vesicle trafficking function in sperm acrosome formation [15], but its role in auxiliary germ cells such as Sertoli cells has not yet been carried out.

Notably, patients with oligonasthenospermia carrying *PCIK1* deletion mutations in our study showed impaired inhibin B secretion, which may serve as a direct marker of spermatogenesis for sperm count and testicular volume in male infertility patients [32]. It is well known that inhibin B is secreted specifically by Sertoli cells, whose function is characterized by secretion, so we hypothesized that *PICK1* truncating mutations caused impaired vesicular trafficking function. In our study, both truncating mutations could result in the deletion of the BAR functional domain of PICK1 protein. BAR domains are positively charged crescent-shaped modules that mediate the curvature of negatively charged lipid membranes during remodelling processes. As a BAR domain protein, the absence of PICK1 interferes with the formation of secretory particles and the stress response of the endoplasmic reticulum, resulting in a decrease in the number of dense nuclear secretory particles [33, 34]. Holst et al. confirmed a prominent reduction in secretory vesicle number in *PICK1* deficient mouse [35]. In our study, cell function experiments showed the specific cytokines secreted by Sertoli cells in *PICK1* KO mouse were significantly reduced, including BMP4, FGF2, BMPR1B, Cludin, Cdh2 and SOX9, which provided convincing evidence that the vesicle secretion function of Sertoli cells was impaired.

To further confirm the hypothesis that the secretory function of Sertoli cells was impaired due to *PICK1* deletion, we performed multi-omics analyses of *PICK1* KO mouse to provide a noninvasive and efficient assessment. The majority of the microRNAs and mRNAs identified by transcription sequencing have been reported to play an important role in the development of the male reproductive system, verifying the accuracy of our analysis results. Among the six differentially expressed microRNAs, miR-182-5p has been reported to constitutea regulatory network with CRISP3 in the seminal plasma fluid of teratozoospermia patients, indicating miR-182-5p could be a possible biomarker for teratozoospermia [36]. Let-7a-1-3p is an immature precursor of the miR-let-7 family, which is the most abundant miRNA family present in the testis and may be involved in the regulation of cell cycle, proliferation, estrogen and androgen signaling [37, 38].

Interestingly, about a quarter of the differential genes were enriched in vesicle secretion function, such as Apoe, Rab3b and Alpn. ApoE domain structure and polymorphism impacted the kinetics of phospholipid vesicle solubilisation [39]. Rab GTPases are key regulators of membrane trafficking. Apln can regulate the secretion of reproductive hormones and the maturation of germ cells [40]. In addition, the expression levels of WNT4 and Cyp21a1, which are regulator of hormone levels, were upregulated 3 to 5 fold. Increased WNT4 levels have been confirmed to induce germ cell failure [41, 42]. Cyp21a1 affects the concentration of testosterone in the blood [43]. Another remarkable DEG, Atp1a1, has been reported to be universally expressed in Sertoli cells, mediating Sertoli tight junctions and gap junctions through the Src-EGFR-ERK1/2-CREB pathway [44]. Cytoskeletal track selection during cargo transport in spermatids is relevant to male fertility [45]. Another core gene is PTPN11, which is vital in the PPI network. Naturally occurring mutations in PTPN11 caused genetic disorders characterized by a spectrum of defects, including male infertility [38].

Gene function enrichment analysis based on microRNAs and mRNAs highlighted the ‘regulation of vesicle-mediated transport’ and ‘negative regulation of secretion’ pathways. Sertoli cells are crucial for spermatogenesis in the seminiferous epithelium because their actin cytoskeleton supports vesicular trafficking, cell junction formation, protein anchoring, and spermiation [46]. In addition, vesicle-associated membrane protein-associated protein in Sertoli cells has been confirmed to be involved in androgen receptor trafficking [47]. The secretion of inhibin B in male infertility patients and subsequent enrichment analysis of biological function suggested that *PICK1* deficiency may affect the differentiation and maturation of sperm by destroying the vesicle trafficking of Sertoli cells, thus leading to male infertility. Sertoli cells of *PICK1* KO mouse were cultured in vitro, and the secretion of various growth factors was reduced, further confirming the previous clinical phenomenon and the results of bioinformatics analysis.

Metabolome analysis with high efficiency, mild injury and diverse samples is able to detect abnormalities that cannot be found by routine semen analysis [48]. Multi-omics analysis combining genomics and metabolomics can improve diagnostic efficiency and accuracy [49]. In *PICK1* KO mouse, the significantly increased metabolic molecules were mainly enriched in amino acid metabolism, steroidal hormone biosynthesis and vitamin metabolic pathways. In our study, D-(-)-glutamine, vitamin B12, meaquinone (vitamin K2), and beta-estradiol 17-acetate could serve as metabolic biomarkers for male infertility caused by *PICK1* deletion. Recent metabolism studies of male infertility have confirmed that vitamin metabolism dysfunction can lead to delayed differentiation of spermatogonium [50]. Most of the energy required for Sertoli cells is derived from single oxidation of glutamine [51], the expression of which was significantly elevated in nonobstructive azoospermia and asthenospermia patients compared with healthy controls [52]. In this study, among all the differentially expressed metabolites, the alteration of D-(-)-glutamine expression was the mostobvious, suggesting glutamine metabolism in the testes of *PICK1* KO mouse was abnormal. Subsequently, abnormal glutamine metabolism may further impair the supply of energy and nutrients by Sertoli cells to germ cells. In the ‘Environmental Information Processing’ category, differential expressed metabolism was enriched in ‘Membrane transport’, suggesting impaired vesicle trafficking. Moreover, metabolomics analysis suggests abnormalities in the endocrine system, which is a diagnosis and treatment target for male infertility [53].

## Conclusion

In summary, we performed an exome study to genetically dissect samples from male infertility patients with Sertoli cell dysfunction and identified two loss-of-function deletion mutations in *PICK1*. By combining multiple biological annotations and functional experiments, we demonstrated that *PICK1* deletion induced male infertility through impaired secretory function of Sertoli cells. Based on integrated multi-omics analysis, we identified a variety of significantly differentially expressed molecules that could be candidate biomarkers for male infertility induced by *PICK1* deletion.

### Electronic supplementary material

Below is the link to the electronic supplementary material.

Supplementary Material 1

## Data Availability

The datasets used and analyzed during the current study are available from the corresponding author on reasonable request.

## Abbreviations

BAR: Bin-Amphiphysin-Rvs
WES: Whole-exome sequencing
PICK1: Protein interacting with C-kinase 1
BMP4: Bone morphogenetic protein 4
FGF2: Fibroblast growth factor 2
AR: Androgen receptor
PCA: Principal component analysis
BP: Biological process
KEGG: Kyoto Encyclopedia of Genes and Genomes
PPI: Protein–protein interaction
DEGs: Differentially expressed genes
CKO: Conditional knock out
WT: Wide type
