# Supplementary material for: Multi-omics study identifies that PICK1 deficiency causes male infertility by inhibiting vesicle trafficking in Sertoli cells
Source: Reprod Biol Endocrinol. 2023 Nov 25;21:114. doi: 10.1186/s12958-023-01163-w (PMC10675906; doi:10.1186/s12958-023-01163-w)
Supplement: Supplementary file 1 — Supplementary Material 1 [file 12958_2023_1163_MOESM1_ESM.docx]

**Supplemental Figures**


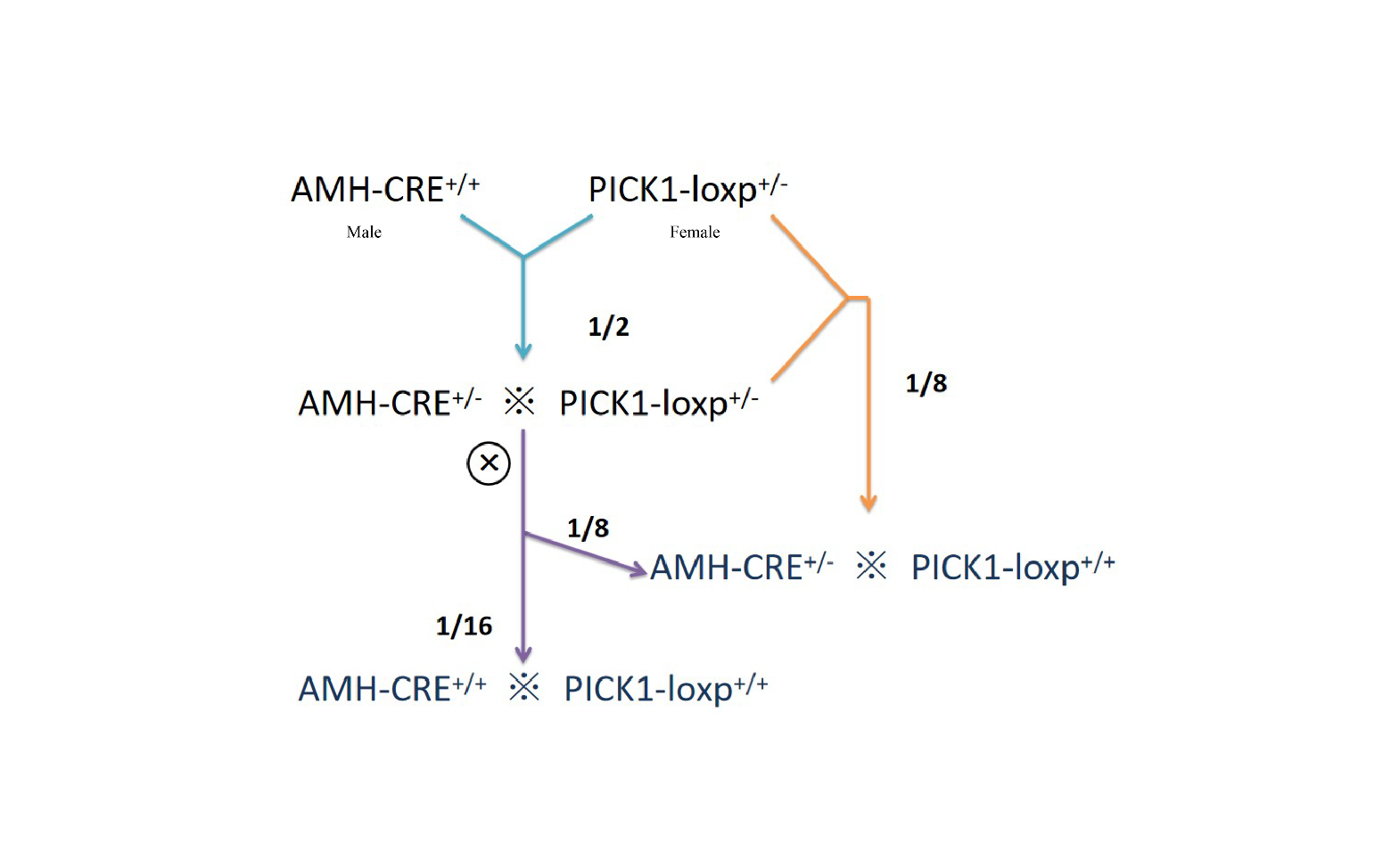


**Supplemental Figure1. The construction process of Sertoli cell specific PCIK1 knockout (CKO) mice.**

**
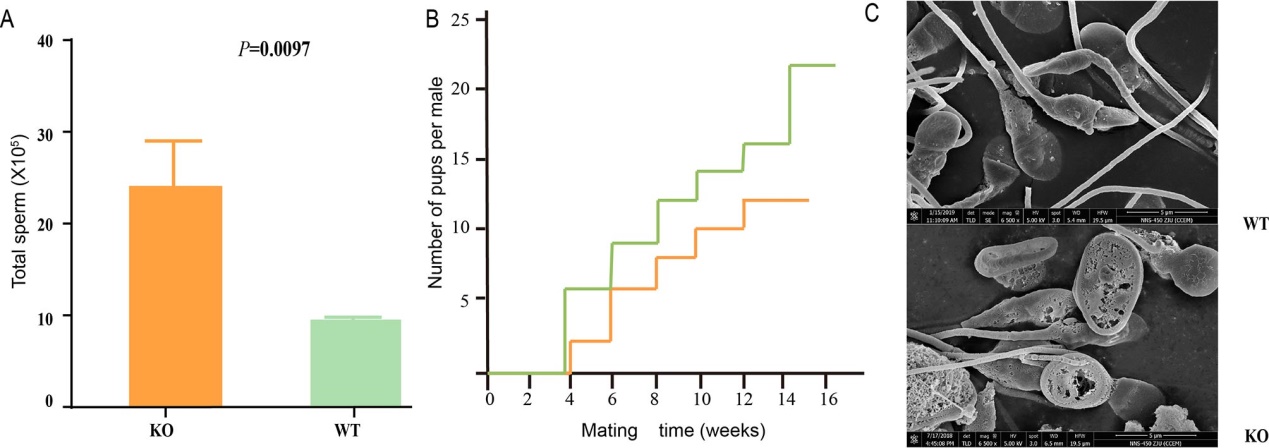
**

**Supplemental Figure2. Evaluation of PICK1 KO mouse fertility.** The total sperm counts of *PICK1* KO mouse and WT mouse (Figure S2A). Breeding experiments of *PICK1* KO mouse and WT mouse (Figure S2B). Morphology of sperm from *PICK1* KO mouse and WT mouse under electron microscope (Figure S2C).


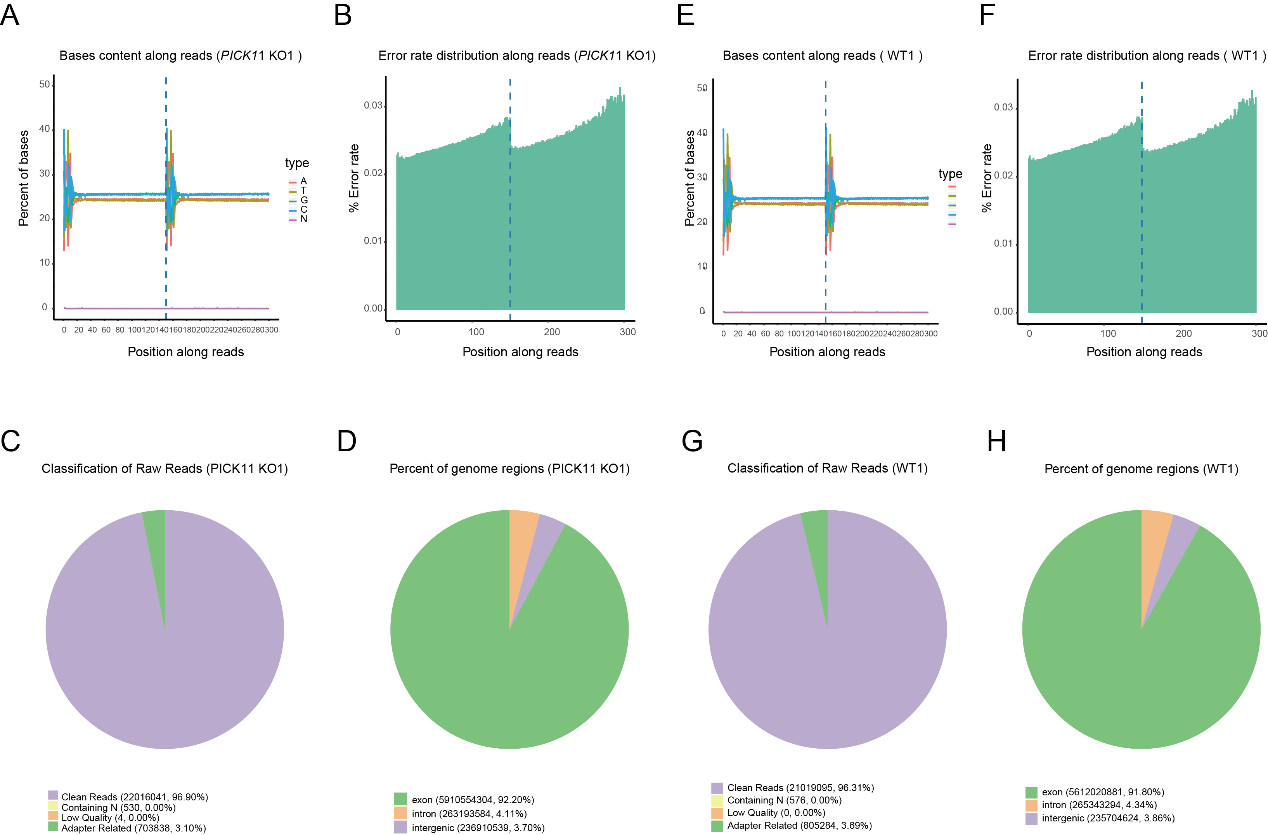


**Supplemental Figure3. Quality control of raw data and result of genome map.** The quality control analysis of the raw date of PICK1 KO group (Figure S3A-3D) and WT group (Figure S3E-3H).


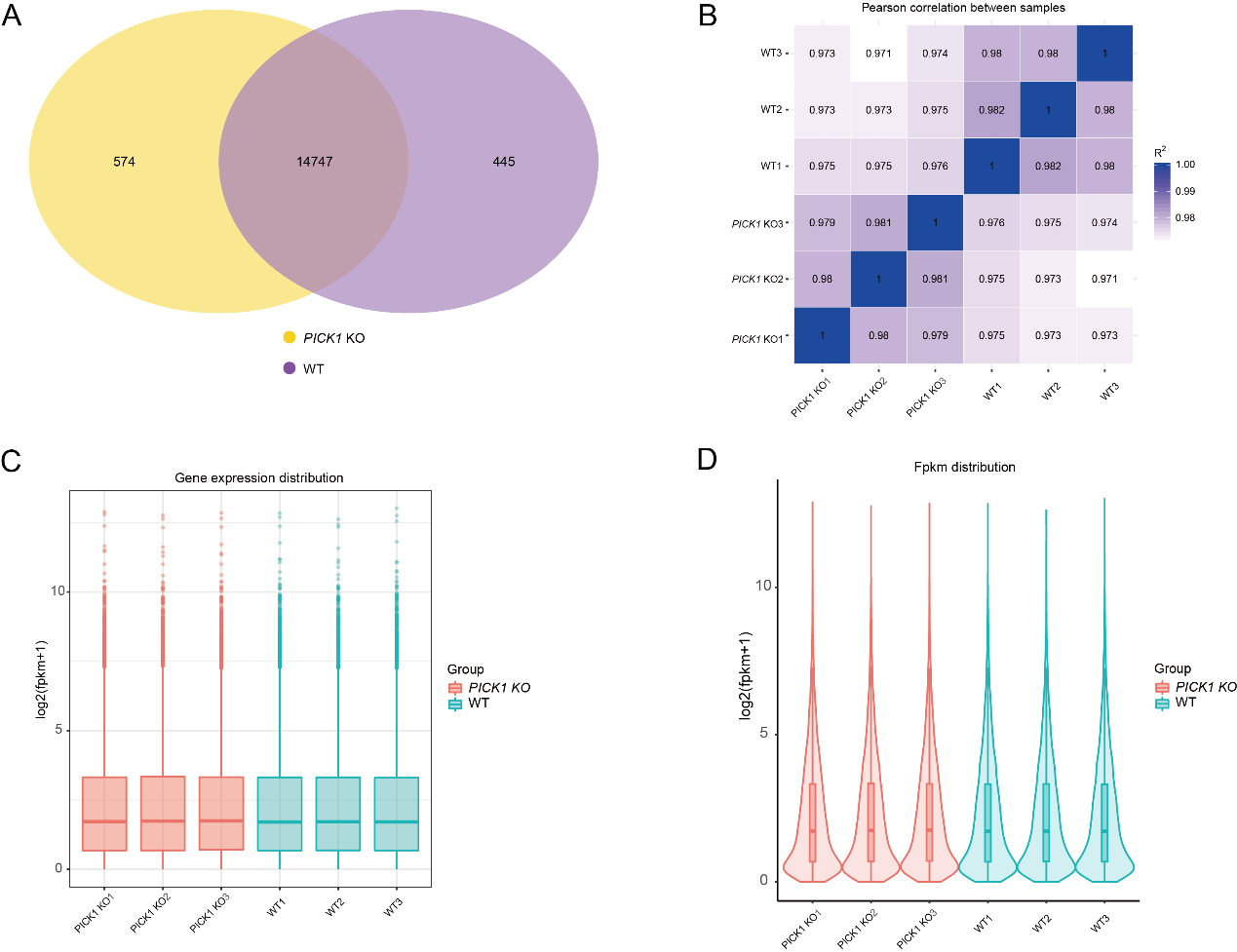


**Supplemental Figure4. The quant results of gene expression.** The Venn diagram showed 14,747 genes were expressed in both the KO group and WT group (Figure S4A) and Pearson correlation analysis between sample showed the inter-group differences and intra-group consistency (Figure S4B). The quant results of gene expression showed the homogeneity of gene expression abundance (Figure S4C, D).


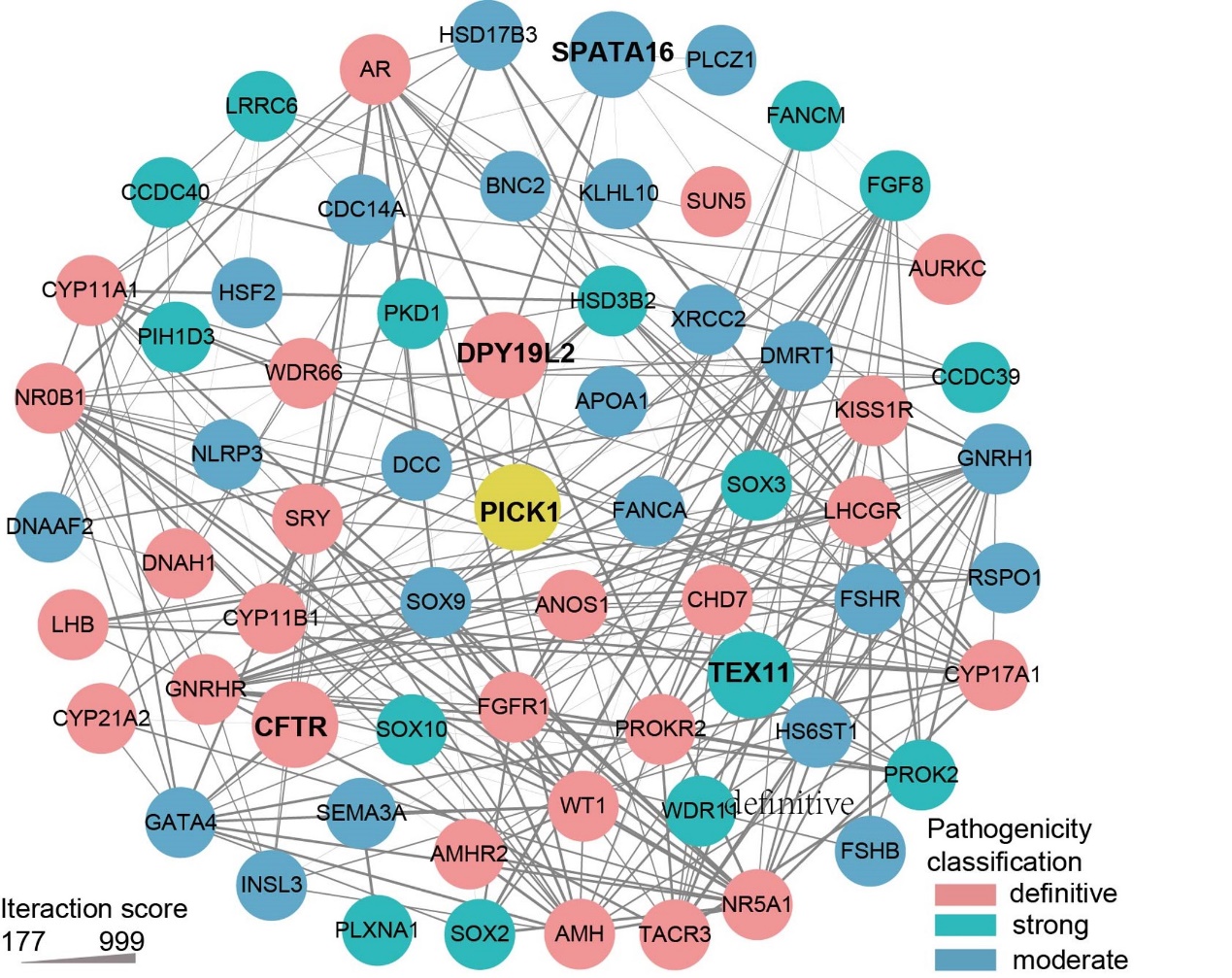


**Supplemental Figure5. The PPI network of PICK1 and explicit male infertility genes.** PICK1 interacted with 25 definitive genes and 15 strong genes.
